# Pattern of Live Births in Rio de Janeiro State, Brazil, According to Robson Groups and the Kotelchuck Index Classification - 2015/2016

**DOI:** 10.1055/s-0040-1712122

**Published:** 2020-07

**Authors:** Luciana Leite de Mattos Alcantara, Núbia Karla de Oliveira Almeida, Renan Moritz Varnier Rodrigues de Almeida

**Affiliations:** 1 Programa de Engenharia Biomédica, Universidade Federal do Rio de Janeiro, Rio de Janeiro, RJ, Brazil; 2 Departamento de Estatística, Universidade Federal Fluminense, Niterói, RJ, Brazil

**Keywords:** vaginal birth, c-sections, kotelchuck index, robson classification, parto vaginal, cesárea, índice de kotelchuck, classificação de robson

## Abstract

**Objective** To investigate the patterns of hospital births in the state of Rio de Janeiro (RJ), Brazil, between 2015 and 2016; considering the classification of obstetric characteristics proposed by Robson and the prenatal care index proposed by Kotelchuck.

**Methods** Data obtained from the Information System on Live Births of the Informatics Department of the Brazilian Unified Health System (SINASC/DATASUS, in the Portuguese acronym) databases were used to group pregnant women relatively to the Robson classification. A descriptive analysis was performed for each Robson group, considering the variables: maternal age, marital status, schooling, parity, Kotelchuck prenatal adequacy index and gestational age. A logistic model estimated odds ratios (ORs) for cesarean sections (C-sections), considering the aforementioned variables.

**Results** Out of the 456,089 live births in Rio de Janeiro state between 2015 and 2016, 391,961 records were retained, 60.3% of which were C-sections. Most pregnant women (58.6%) were classified in groups 5, 2 or 3. The percentage of C-sections in the Robson groups 1, 2, 3, 4, 5 and 8 was much higher than expected. Prenatal care proved to be inadequate for women who subsequently had a vaginal delivery, had an unfavorable family structure and a lower socioeconomic status (mothers without partners and with lower schooling), compared with those undergoing cesarean delivery. For a same Robson group, the chance of C-section increases when maternal age rises (OR = 3.33 for 41–45 years old), there is the presence of a partner (OR = 1.81) and prenatal care improves (OR = 3.19 for “adequate plus”).

**Conclusion** There are indications that in the state of RJ, from 2015 to 2016, many cesarean deliveries were performed due to nonclinical factors.

## Resumo

**Objetivo** Investigar os padrões dos partos hospitalares no estado do Rio de Janeiro (RJ), Brasil, entre 2015 e 2016, considerando a classificação de características obstétricas de Robson e a dos cuidados pré-natais proposta por Kotelchuck.

**Métodos** Dados sistema de informações sobre nascidos vivos (SINASC) do departamento de informática do sistema único de saúde (DATASUS) foram utilizados para agrupar gestantes relativamente à classificação de Robson. Foi efetuada uma análise descritiva para cada grupo de Robson, considerando-se as variáveis idade materna, estado civil, escolaridade, paridade, o índice de Kotelchuck de adequação do pré-natal e a idade gestacional. Também foi realizado o cálculo de razão de chances (RC) para parto cesáreo, considerando-se um modelo logístico.

**Resultados** Dos 456.089 nascimentos vivos ocorridos no RJ de 2015 a 2016, foram incluídos 391.961 registros, sendo 60,3% cesáreas, com maioria de gestantes (58,6%) nos grupos 5, 2 ou 3. O percentual de cesáreas nos grupos 1, 2, 3, 4, 5 e 8 foi bem superior ao sugerido pela literatura. Para gestantes de um mesmo grupo (controladas as demais características), a chance de cesárea se eleva quando aumenta a idade materna (RC = 3,33 para 41–45 anos), existe a presença de um companheiro (RC = 1,81), o nível de escolaridade é maior (RC = 3,11 para ≥ 12 anos) e o pré-natal é mais cuidadoso (RC = 3,19 para “adequado plus”).

**Conclusão** Há indícios que no RJ, de 2015 a 2016, muitos partos cesáreos foram realizados sob influência de fatores extraclínicos.

## Introduction

Cesarean sections (C-sections) are effective interventions for the protection of maternal and neonatal life,^1^ but should only be performed for well-defined clinical reasons. Cesarean sections can cause significant and sometimes permanent complications, as well as sequelae or death, especially when the infrastructure and/or the ability to safely perform the procedure and treat its postoperative complications is lacking. Therefore, a cesarean delivery should be individualized and only performed when clinically necessary, that is, for pregnant women with full-centered or partial placenta, HIV-carriers, or for pregnant women with systemic uterine rupture.^2^ ^3^

However, the association of birth delivery and nonclinical factors is well known, and, for instance, pregnant women with higher education end to be more frequently submitted to cesareans.^4^ ^5^ ^6^ In addition, in Brazil, cesarean deliveries in private sector facilities (privately-owned, either government or private sector insured) is much higher than in public health facilities.^7^ ^8^ Also, a positive association between the socioeconomic status of mothers and cesarean section rates has been detected in studies, especially among women who had private health services, possibly due to the scarcity of options for vaginal delivery in the private health sector.^8^ Therefore, socioeconomic status seems to be an important predictor of C-sections.

In 1985, the World Health Organization (WHO)^9^ recommended that the percentage of cesarean sections should not exceed 15% of all deliveries, as studies have shown no association between an increase in cesarean rates above these values and a reduction in mortality. Moreover, in 2014, the WHO conducted an ecological study using the latest world data and concluded that a substantial part of the association between cesarean rates and mortality was explained by socioeconomic factors, as well as estimating that cesarean rates of > 10% had no effect on mortality.^1^

Therefore, in 2015, the WHO decided to reevaluate the appropriate value for C-section levels, but found no reliable and internationally accepted classification system for delivery monitoring. The WHO then proposed, after conducting a systematic review of existing systems, that the Robson Classification (see below) should be used as a standard instrument to assess, monitor and compare cesarean rates, either along time in a same cohort or cross-sectionally, for instance in a set of hospitals.^1^

However, despite these WHO concerns, the rate of cesarean deliveries has been steadily increasing in the world, and in Brazil, in 1994, 2004 and 2014, this percentage reached 32.02%, 41.75% and 56.99%, respectively (close to an 80% increase in 20 years).^10^ Thus, there is a need for a greater understanding of the factors interfering with delivery modality, so as to reduce the number of cesareans without adequate clinical indication. With this aim, the present study investigated the patterns of hospital deliveries in the state of Rio de Janeiro (RJ), Brazil, between 2015 and 2016. Maternal obstetric characteristics were categorized according to the groups proposed by Robson and were evaluated in relation to maternal, gestational and delivery characteristics.

## Methods

As mentioned, to monitor, evaluate and compare cesarean rates, the WHO proposed the adoption of the Robson classification system,^11^ which categorizes pregnant women into 10 major groups. These groups are created from five characteristics available at the time of delivery: parity, onset of birth, gestational age, fetal presentation and number of fetuses (Table 1).^12^ The Robson Classification has the advantages of being at the same time “inclusive” and “mutually exclusive.”^13^ Inclusive, in the sense that all assisted women will be included in the classification, a very important characteristic for prospective data analysis; and mutually exclusive, since each woman is classified in one and only one group. The Robson Classification, in this manner, helps to answer who are the women undergoing cesarean section, and therefore could help to explain if there are cesarean excesses in a specific group.^1^ In line with the WHO proposal, the Brazil Declaration of Live Births (DN, in the Portuguese acronym) form, starting in 2011, records information which allows the determination of these groups.^14^

**Table 1:** Robson groups classification

	Robson groups									
Obstetric characteristics	1	2	3	4	5	6	7	8	9	10
Obstetric history										
Nuliparous	X	X				X		X	X	X
Multiparous, no previous C-section			X	X			X	X	X	X
Multiparous, previous C-section					X		X	X	X	X
Number of fetus										
1	X	X	X	X	X	X	X		X	X
≥ 2								X		
Fetal presentation										
Cephalic	X	X	X	X	X			X		X
Pelvic						X	X	X		
Transversal or oblique								X	X	
Gestational age (weeks)										
Preterm (< 37)						X	X	X	X	X
Term (≥ 37)	X	X	X	X	X	X	X	X	X	
Delivery										
Spontaneous	X		X		X	X	X	X	X	X
Induction or C-section prior to delivery		X		X	X	X	X	X	X	X

**Source:** Robson.^12^

The Kotelchuck index (KC),^15^ on the other hand, assesses the quality of prenatal care by calculating the percentage of consultations performed among those that would be expected to do so, relative to gestational age. Prenatal care is then classified into 1 of 4 categories: inadequate (< 50%), intermediate (50% to 79%), adequate (80% to 109%) and adequate plus (≥ 110%). In the present study, the category “not done” was added to the categories above.

The present study used records of hospital live births in the state of Rio de Janeiro, Brazil, between 2015 and 2016, fetuses without anomalies and mothers aged between 15 and 45 years old. Data were obtained from the country Information System on Live Births of the Informatics Department of the Brazilian Unified Health System (SINASC/ DATASUS, in the Portuguese acronym).^14^ The following variables were collected: maternal age (years old), marital status, schooling (years), parity, KC index of prenatal care (already available in the database), gestational age (weeks) and weight of the newborn (grams).The database already had a “Robson classification” variable assigned to cases. However, the quality of this variable was not considered as good enough, due to a large number of missing/inconsistent data. Therefore, this variable was recalculated by the present researchers, taking into account other information available in the database.

Only records of hospital births with complete information and without data inconsistences were retained in the study. Records were excluded when any of the following criteria was not met:

1) Maternal age ≤ 16 years and mother with < 12 years of study;
2) Total previous pregnancies ≤ maternal age - 13; or
3) Total previous pregnancies ≤ number of live births + number of dead children born.

Newborns with congenital anomalies were also excluded. References for adequate cesarean proportions according to group were obtained from the literature.^16^

A logistic model was estimated using birth modality (vaginal versus C-section) as the dependent variable, and, as predictors, the variables age, marital status, schooling level, the KC index and the Robson group of the women. Odds ratios (ORs) and their 95% confidence intervals (CIs) were estimated for all variables in this model.^17^ All data processing and analysis was performed with the help of IBM SPSS Statistics for Windows, Version 20.0 (IBM Corp., Armonk, NY, USA)

## Results

After exclusion of records without complete information or with inconsistent data, out of the 456,089 live births in the state of Rio de Janeiro between 2015 and 2016, 391,961 records could be retained. It could be seen that 77.8% of the pregnant women were between 18 and 34 years old, 41.6% were nulliparous, 35.0% had a partner, 19.4% had > 11 years of schooling, 87.1% had a full term gestation (37 - 41 weeks), 71.8% had a prenatal score classified as adequate or adequate plus and only 39.7% had vaginal delivery (a much lower frequency than the expected 70–75%).

Tables 1 and 2 present the distribution of births in RJ according to the Robson groups, as well as the C-section percentages in each group and their contribution to the cesarean total. The percentage of births in groups 1 and 2 (35.6%) is in agreement with expected values (35.0% to 42.0%). Groups 3, 4, 6 and 7 also had acceptable distributions, but groups 5, 8 and 9 exceeded the expected limits. Still considering Table 3, the highest proportion of births (23.0%) was observed in Robson group 5 (multiparous pregnant women with previous cesarean section), although groups 1, 2 and 3 also had high numbers. Regarding the percentage of cesareans according to group, the high values in groups 1 and 2 (nulliparous women who had a full-term pregnancy), and in group 1 (spontaneous delivery) are noteworthy. Also the high percentage of cesareans in group 10, that is, with preterm gestation, is interesting. The groups that contributed the most to cesarean deliveries were 5 and 2 (the latter includes previous induction of labor or cesarean section). In the last column, one may notice that the total contribution of groups 1, 2 and 5 (56.1%), is in accordance with the expected “no more than 2/3 of cesareans” for these groups (Tables 1 and 2).^12^

**Table 2:** Distribution of births according to Robson groups, cesarean section according to Robson groups and Robson groups contribution in cesarean sections *n* = 391,961

	Cases		Cesarean section in group		Group contribution in cesarean sections (n = 236,306)
Robson group	Observed	Expected	Observed	Expected	Observed
	n(%)	%	%	%	%
1	68,887 (17.6)	35.0–42.0	45.6	10.0	13.3
2	70,636 (18.0)		72.9	25.0–30.0	21.8
3	64,121 (16.4)	30.0–40.0	20.1	3.0	5.5
4	41,556 (10.6)		52.8	20.0	9.3
5	90,266 (23.0)	10.0	86.3	50.0–60.0	33.0
6	5,292 (1.4)	< 5.0	93.2	—	2.1
7	6,492 (1.7)		88.8	—	2.4
8	8,541 (2.2)	1.5–2.0	87.5	60.0	3.2
9	855 (0.2)	0.2–0.6	97.3	100.0	0.4
10	35,315 (9.0)	4.0–5.0	61.4	15.0–20.0	9.2

**Source:** Robson.^12^

**Table 3:** Distribution of births according to pregnant characteristics and by Robson group for each mode of delivery *n* = 391,961

Characteristics	Categories	N	%	1	2	3	4	5	6	7	8	9	10
**Cesarean section (n = 236,306)**													
Age (years old)	15 to 17	10,630	4.5	11.2	7.5	2.2	1.4	0.9	9.4	1.2	3.3	4.0	5.2
	18 to 20	23,459	9.9	18.5	13.9	7.6	5.5	5.6	14.6	5.6	8.0	7.7	10.4
	21 to 34	158,434	67.0	62.2	67.5	70.0	69.1	69.4	62.7	64.9	66.0	57.3	63.1
	35 to 40	38,458	16.3	7.5	10.1	17.3	20.7	21.1	12.1	24.1	20.2	25.0	18.2
	41 to 45	5,325	2.3	0.6	1.0	2.9	3.2	3.0	1.3	4.2	2.5	6.0	3.2
	Percentile 25	23	20	21	24	25	25	21	25	24	25	23	
	Percentile 50	28	24	26	29	30	30	26	31	30	31	29	
	Percentile 75	33	30	31	33	34	34	32	35	34	35.75	34	
Marital status	Without a partner	130,684	55.3	58.8	51.8	63.2	58.2	53.4	54.1	58.0	55.6	53.5	57.4
Schooling (years)	0	224	0.1	0.0	0.0	0.3	0.1	0.1	0.0	0.2	0.1	0.1	0.1
	1 to 3	3,039	1.3	0.6	0.5	2.5	1.7	1.6	0.5	2.5	1.6	1.6	1.6
	4 to 7	31,715	13.4	10.9	7.1	19.9	15.6	16.2	10.3	19.2	13.9	13.0	15.1
	8 to 11	137,054	58.0	60.6	53.8	63.2	62.2	58.5	52.9	56.6	55.1	49.9	57.9
	12 or +	64,274	27.2	27.9	38.6	14.1	20.4	23.6	36.2	21.6	29.2	35.5	25.2
Parity	Nulliparous	99,840	42.3	100.0	100.0	0.0	0.0	0.0	100.0	0.0	39.3	37.6	40.4
Prenatal (KC Index)	Not done	1,187	0.5	0.3	0.1	0.6	0.4	0.6	0.6	1.2	0.6	1.1	1.0
	Inadequate	38,454	16.3	14.1	11.0	17.9	15.7	19.3	14.4	19.0	18.1	18.3	19.6
	Intermediate	11,381	4.8	3.8	2.5	5.1	4.1	4.5	5.5	7.3	7.0	6.1	11.7
	Adequate	16,469	7.0	6.4	5.1	8.0	7.3	7.0	6.0	7.1	7.9	5.3	11.1
	Adequate Plus	168,815	71.4	75.3	81.2	68.3	72.4	68.6	73.5	65.4	66.5	69.2	56.7
Gestational age (weeks)	< 37	27,800	11.8	0.0	0.0	0.0	0.0	0.0	16,2	17.6	54.4	28.6	100
	37	24,328	10.3	10.7	11.0	10.9	11.7	11.0	11.3	11.5	19.7	12.9	0.0
	38	60,516	25.6	26.3	31.3	24.6	29.1	28.9	26.8	24.7	15.6	18.6	0.0
	39	71,350	30.2	31.8	34.7	33.2	37.3	35.4	26.1	26.7	7.2	18.9	0.0
	40	33,345	14.1	19.6	14.9	18.9	13.9	15.9	13.3	12.8	2.4	14.2	0.0
	41	14,265	6.0	9.0	6.2	9.3	5.8	6.7	4.6	5.0	0.5	5.3	0.0
	> 41	4,702	2.0	2.8	2.0	3.0	2.2	2.2	1.8	1.8	0.3	1.6	0.0
	Mean	38.29	39.00	38.81	39.02	38.81	38.86	37.92	37.81	35.69	37.10	34.23	
Weight (grams)	≥ 2,500	90.6	96.1	96.3	96.2	96.5	97.4	85.3	85.9	42.7	73.7	55.2	
**Vaginal delivery (n = 155,655)**													
Age (years)	15 to 17	18,749	12.0	24.9	23.5	3.3	3.3	2.1	22.4	2.3	6.3	13.0	15.6
	18 to 20	31,212	20.1	30.2	29.6	14.0	13.5	11.1	20.2	10.2	13.9	17.4	20.0
	21 to 34	92,157	59.2	42.4	44.4	70.4	70.8	73.0	53.5	67.4	63.3	60.9	54.1
	35 to 40	11,918	7.7	2.3	2.2	10.7	11.0	12.1	3.6	16.8	15.0	4.3	8.8
	41 to 45	1,619	1.0	0.1	0.2	1.5	1.5	1.7	0.3	3.3	1.5	4.3	1.5
	Percentile 25	19	18	18	22	22	22	18	23	22	19	19	
	Percentile 50	23	20	20	26	26	27	21	28	26	25	23	
	Percentile 75	29	24	24	31	31	32	27	33	32	29	29	
Marital status	Without a partner	31,615	79.7	81.4	80.7	79.0	79.1	76.1	73.7	73.0	76.4	69.6	80.9
Schooling (years)	0	280	0.2	0.1	0.0	0.3	0.2	0.3	0.0	0.3	0.3	0.0	0.2
	1 to 3	3,616	2.3	0.9	0.9	3.2	2.9	3.4	1.7	4.4	3.1	0.0	2.8
	4 to 7	38,927	25.0	18.2	18.9	29.4	29.7	27.8	14.1	27.1	29.6	26.1	26.4
	8 to 11	101,179	65.0	70.0	70.2	62.4	62.4	61.1	69.0	59.1	55.0	56.5	62.3
	12 or +	11,653	7.5	10.8	10.1	4.8	4.8	7.5	15.2	9.1	12.0	17.4	8.2
Parity	Nulliparous	63,270	40.6	100.0	100.0	0.0	0.0	0.0	100.0	0.0	27.8	60.9	43.8
Prenatal (KC Index)	Not done	2,715	1.7	0.8	0.4	2.4	1.2	2.5	3.3	6.3	2.7	4.3	3.5
	Inadequate	44,704	28.7	23.8	22.5	31.9	30.9	32.3	21.6	28.4	31.4	39.1	32.8
	Intermediate	11,931	7.7	5.8	6.1	7.0	7.4	7.5	18.0	15.3	17.9	8.7	16.4
	Adequate	12,567	8.1	7.5	8.1	7.5	7.9	8.1	7.5	8.3	9.1	0.0	12.0
	Adequate Plus	83,738	53.8	62.2	62.8	51.2	52.6	49.6	49.6	41.7	38.9	47.8	35.3
Gestational age (weeks)	< 37	14,655	9.5	0.0	0.0	0.0	0.0	0.0	34.9	27.6	65.3	34.7	100
	37	12,434	8.0	9.5	8.3	8.6	8.3	8.5	8.6	9.1	11.4	13.0	0.0
	38	27,404	17.6	20.3	18.6	19.4	18.2	19.3	13.6	17.4	13.8	17.4	0.0
	39	49,624	31.9	34.4	36.3	34.7	36.8	35.9	22.2	25.6	5.7	13.0	0.0
	40	35,456	22.8	25.1	24.0	25.9	24.6	25.8	17.7	13.6	2.5	17.4	0.0
	41	12,526	8.0	8.2	10.5	8.7	9.7	8.3	2.8	5.1	0.7	4.3	0.0
	> 41	3,556	2.3	2.4	2.3	2.8	2.4	2.1	0.3	1.7	0.6	0.0	0.0
	Mean	38.63	39.11	39.18	39.17	39.18	39.14	35.44	36.38	37.73	34.83	33.84	
Weight (grams)	≥ 2,500	92.3	95.4	96.0	97.0	97.0	96.7	64.3	71.5	28.9	60.9	56.6	

Table 3 presents the distribution of births according to, respectively, the characteristics of the pregnant woman and the gestation, by Robson group and by mode of delivery. A total of 42.3% of the women who underwent cesareans were nulliparous, a percentage not very different from that observed among those who had vaginal delivery (40.6%). Regardless of the Robson group, most cesarean sections were performed in pregnant women with a partner and > 11 years of schooling (Table 3). Regarding gestational characteristics (Table 3), the percentage of women who did not perform/had an inadequate prenatal care was significantly higher for vaginal delivery, whatever the Robson group.

Table 4 presents the percentages of cesarean sections by obstetric characteristics and estimated (ORs) for cesarean delivery obtained through the logistic model. For women in a same Robson group, the chance of cesarean delivery rises if maternal age increases (OR = 3.33 for 41–45 years old), if a partner is present (OR = 1.81), if the level of schooling is higher (similar odds for levels of 0, 1–3 and 4–7 years; OR = 3.11 for 12+ years of study) and if prenatal care improves (similar odds for the categories “inadequate” and “intermediate”; OR = 3.19 for “adequate plus”).

**Table 4:** Frequency of cesarean section by obstetrics characteristics; odds ratios and respective 95% Confidence Intervals for caesarean section *n* = 391,961

Characteristics	% of cesarean section	OR	95% CI
Age (years old)			
15 to 17	36.2	1	
18 to 20	42.9	1.18	1.14–1.21
21 to 34	63.2	1.87	1.84–1.90
35 to 40	76.3	2.85	2.81–2.89
41 to 45	76.7	3.33	3.26–3.41
Marital status			
Without a partner	51.3	1	
With a partner	77.0	1.81	1.79–1.83
Schooling (years)			
0	44.4	1	
1 to 3	45.7	1.02	0.80–1.24
4 to 7	44.9	1.13	0.92–1.34
8 to 11	57.5	1.45	1.24–1.66
12 or +	84.7	3.11	2.90–3.33
Prenatal (KC index)			
Not done	30.4	1	
Inadequate	46.2	1.92	1.83–2.00
Intermediate	48.8	1.92	1.84–2.01
Adequate	56.7	2.60	2.52–2.69
Adequate Plus	66.8	3.19	3.11–3.27
Robson's group			
1	45.6	1	
2	72.9	2.79	2.76–2.81
3	20.1	0.28	0.26–0.31
4	52.8	1.17	1.14–1.20
5	86.3	6.45	6.42–6.48
6	93.2	15.49	15.38–15.60
7	88.8	8.54	8.46–8.62
8	87.5	7.46	7.39–7.53
9	97.3	37.41	36.99–37.83
10	61.4	1.92	1.89–1.95

Abbreviations: CI, confidence interval; OR, odds ratio.

## Discussion

The present study aimed to investigate the patterns of hospital births in the state of Rio de Janeiro, Brazil, with a specific interest in the occurrence of C-sections versus vaginal births. Pregnant women were analyzed according to their classification into Robson groups.^12^ In this classification, groups 1–2 (nulliparous) and 3–4 (multiparous without previous cesarean section) differ only in the beginning of labor. Group 5 does not make restrictions on the onset of labor and considers only multiparous pregnant women, who already have previous cesarean sections, with a full term pregnancy and with the fetus in the cephalic position. Groups 6 and 7 (single fetus in the pelvic position) are different only with respect to the obstetric history of the woman, group 8 refers to multiple pregnancies, group 9 to those where the fetus is in the transverse or oblique position, and, finally, group 10 concerns only preterm pregnancies with the fetus in the cephalic position.

Although cesarean rates in groups 1–5 (with a less clear clinical indication for a C-section) are much higher than expected (Tables 1 and 2), these results in fact agree with previous studies in the country.^7^ ^13^ ^18^ Therefore, it may be said that cesarean sections actually are being performed electively in the studied population, without strict clinical indications.^7^ ^13^ In addition, it is alarming that in the Robson groups 9 (transverse or oblique fetal position) and 10 (preterm pregnancies) “inadequate”/”not done” prenatal care percentages were, respectively, 43.4% and 36.3% (Table 3). These results are strong evidence of inadequate prenatal care in women with greater obstetric complexity and vaginal delivery,^16^ and probably indicate the influence of socioeconomic factors in the health care of these women.

To our knowledge, this is the first study with such a large dataset that analyses the association of cesarean risk and the level of prenatal care/socio-economic status (e.g., educational level) according to Robson groups. As already mentioned, it was clear from the analysis that C-sections are performed more frequently among women with higher social conditions (according to measures of schooling, prenatal care and marital status). The reasons for this preference are complex and still a matter of debate, but it is well-known that doctors many times influence women towards C-sections, for instance, since surgical procedures allow for a better control of the delivery schedule (day and time).^19^ ^20^

The Robson classification can be very useful for the monitoring and analysis of birth modalities. For instance, in Brazil, a study using the Robson classification detected a need for reducing (elective) cesarean deliveries in nulliparous women, thereby reducing its recurrence in multiparous women.^7^ Another study in the country, also with the help of the Robson 10 group classification, concluded that public policies aimed at raising awareness about avoiding a first C-section and allowing spontaneous labor are necessary for a long-term decrease in C-section rates.^13^

Other interesting comments can be made taking into account the information in Tables 1 2 3 to 4. For example, in group 1 (nulliparous women with spontaneous labor), cesarean section, most women (75%) were < 31 years old (Table 3) and had a mean gestational age of 39 weeks (Table 3). Therefore, despite favorable conditions for vaginal delivery,^4^ young women with a full term pregnancy had a first birth via cesarean delivery. In group 6 (nulliparous with the fetus in pelvic presentation), 93.2% of the births were via C-sections (Table 3), but only 16.2% of these procedures concerned pregnancies with < 37 weeks (Table 4). Therefore, it is possible to suppose that, in many cases, a vaginal delivery would have been possible with the help of an external cephalic version (ECV) procedure. Also, looking at Robson's groups 1 to 9, pregnancies at 37 or 38 weeks are more frequent in cesarean (31.5% to 42.3%) than in vaginal deliveries (22.2% to 30.4%), indicating shorter pregnancies. In group 10 (preterm gestations), among the 61.4% cesarean births (Tables 1 and 2), more than half of the fetuses had weight in the normal range (≥ 2,500 g), another indication of unnecessary surgical procedures (Table 3). Finally, the similar proportions found for cesareans in nulliparous versus vaginal deliveries also indicate poor incentives for vaginal delivery during the first birth. The main strength of the present study is its populational characteristics, with an unusually large and recent dataset available for analysis. Its main limitation concerns the absence, in the database, of other demographic or clinical variables (e.g., hypertension, diabetes) that could add interesting information for the analysis.

## Conclusion

The results strongly suggest that cesarean deliveries are performed excessively in the analyzed dataset, since many pregnant women had favorable clinical conditions for vaginal delivery. The results also seem to indicate the existence of a maximum gestational period, after which a C-section is performed, not primarily for clinical reasons. Concerning prenatal care, a worrying aspect is that the absence of adequate care reached a high percentage of the studied population, and this proportion was even lower among those women who gave birth through the vaginal route.
